# Revealing the Mysteries of Acute Myeloid Leukemia: From Quantitative PCR through Next-Generation Sequencing and Systemic Metabolomic Profiling

**DOI:** 10.3390/jcm11030483

**Published:** 2022-01-18

**Authors:** Cristina Panuzzo, Aleksandar Jovanovski, Muhammad Shahzad Ali, Daniela Cilloni, Barbara Pergolizzi

**Affiliations:** Department of Clinical and Biological Sciences, University of Turin, 10124 Turin, Italy; aleksandar.jovanovski@unito.it (A.J.); muhammadshahzad.ali@unito.it (M.S.A.); barbara.pergolizzi@unito.it (B.P.)

**Keywords:** acute myeloid leukemia (AML), RT-qPCR, MDR, digital droplet PCR (ddPCR), next-generation sequencing (NGS), metabolomics analysis, metabolomic profiling

## Abstract

The efforts made in the last decade regarding the molecular landscape of acute myeloid leukemia (AML) have created the possibility of obtaining patients’ personalized treatment. Indeed, the improvement of accurate diagnosis and precise assessment of minimal residual disease (MRD) increased the number of new markers suitable for novel and targeted therapies. This progress was obtained thanks to the development of molecular techniques starting with real-time quantitative PCR (Rt-qPCR) passing through digital droplet PCR (ddPCR) and next-generation sequencing (NGS) up to the new attractive metabolomic approach. The objective of this surge in technological advances is a better delineation of AML clonal heterogeneity, monitoring patients without disease-specific mutation and designing customized post-remission strategies based on MRD assessment. In this context, metabolomics, which pertains to overall small molecules profiling, emerged as relevant access for risk stratification and targeted therapies improvement. In this review, we performed a detailed overview of the most popular modern methods used in hematological laboratories, pointing out their vital importance for MRD monitoring in order to improve overall survival, early detection of possible relapses and treatment efficacy.

## 1. Introduction 

Acute myeloid leukemia (AML) is a clonal disorder that affects myeloid progenitor cells residing in the bone marrow (BM). This implies altered differentiation with subsequent abnormal proliferation and accumulation of inadequately matured myeloid cells [1,2]. AML arises mostly as a “de novo” neoplasm in healthy individuals; however, secondary forms of AML (sAML) derived from myelodysplastic syndrome (MDS), myeloproliferative neoplasms (MPNs) or therapies-related (i.e., topoisomerases II, radiation or chemotherapy) are probable [3]. From a molecular point of view, diagnosis is involved with a chromosomal translocation involving crucial genes such as t(8:21), resulting in the formation of *RUNX1-RUNX1T1* fusion gene [4] t(15:17), which generates chimeric *PML-RARA* [5] and inv(16), involving the core binding factor β (*CBF-β*) and the smooth muscle myosin heavy chain (SMMHC), *MYH11* [6].

A cytogenetic profile has allowed stratifying cases into groups that are favorable, intermediate and adverse-risk. Complete identification of genetic mutations has enhanced the previous classification, and it has been useful in determining personalized prognosis and risk stratification. Currently, *NPM1*, *FLT-3*, *CEBPA*, *TP53*, *RUNX1* and *ASXL1* are included to define the profile of prognostic-risk groups [6,7].

To date, diagnosis of AML according to the redesigned guidelines of the World Health Organization (WHO) must provide immunophenotypic, cytogenetic and molecular gene panel screening [8]. Moreover, research efforts in the last decade, carried out on large cohorts of AML patients highlighted the pivotal role of signal transduction alterations in promoting the onset of AML, including non-genomic loss of function of tumor suppressors [9,10]. With reference to this, epigenetic and splicing deregulations or mutations have arisen as third-class mutations due to their impact on cellular differentiation and proliferation. *IDH-1*, *IDH-2*, *DNMT3A* and *TET2* emerged in this context [11]. These acquired driver alterations are expressed in more than 40% of AML cases and may be associated with leukemia progression when occurring with other mutations. Currently, they are evaluated in mostly relapsed cases, knowing that new drugs and specific inhibitors are available (i.e., IDH inhibitors and hypomethylating agents) [12,13].

**Figure 1:**
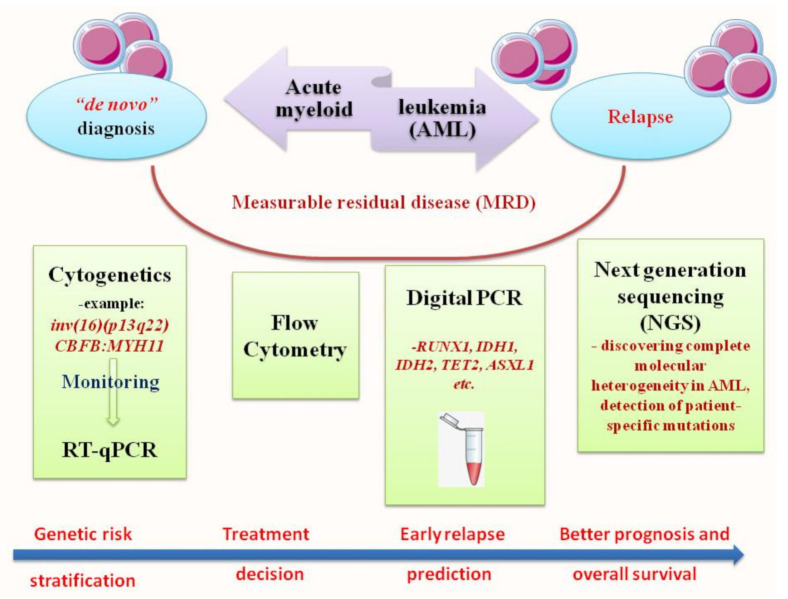
Workflow of diagnostic methods used in “de novo” or relapsed form of AML, as well as its MRD assessment. This scheme highlights the network of methods used in hematology laboratories and the main targets tested, divided into four levels: cytogenetic and RT-qPCR, flow cytometry, digital PCR and next generation sequencing (NGS).

Although ongoing studies have shown improvement in the outcome of AML patients, the real prognosis remains poor. Approximately 70% of patients aged ≥65 died within one year of diagnosis, despite the newly available target treatment options [14]. The molecular identity of AML, enhanced and revised several times from 1976 to 2016, proved its intrinsic genetic complexity. In this direction, diagnosis and prognosis profiles are currently achieved with more sensitive techniques and are used in tandem with canonical methods such as flow cytometry and cytogenetics in order to improve the monitoring of minimal residual disease (MRD), to optimize the treatment, increase overall survival (OS) and to prevent relapse [15,16,17]. In this review, we outlined molecular testing advised for AML diagnosis and measurable residual disease (MRD) assessment in order to translate molecular techniques into clinical practice. Our major focus will be RT-qPCR, digital PCR (ddPCR), next-generation sequencing (NGS) and the recent most attractive approach—metabolomic profiling (Figure 1).

## 2. RT-qPCR: The Gold Standard for Diagnosis and Prognosis Stratification in AML

**Figure 2:**
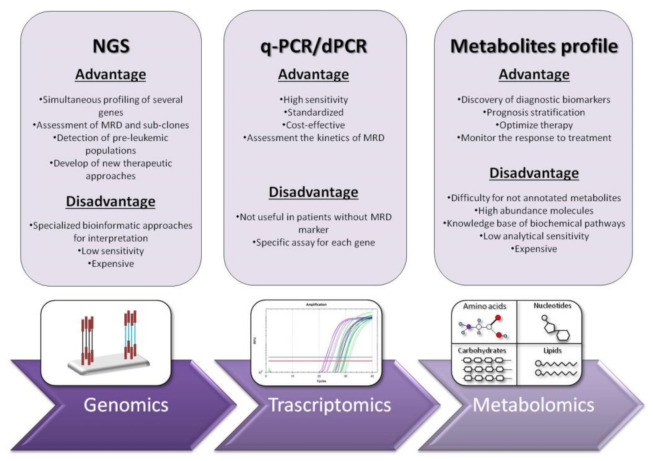
Schematic comparison of “omic” technologies with their main advantages and disadvantages.

The detection of leukemic cells moved in the last two decades from immune-phenotyping to polymerase chain reaction (PCR) and real-time quantitative PCR (RT-qPCR) [18]. RT-qPCR has been consolidated not only for gene expression measurement and common fusion transcripts detection but also for genetic mutations in genes, such as *NPM1*, *FLT3-ITD*, *CEBPA*, *IDH1/2*, *KIT*, *RAS*, *RUNX1* and *TP53* or gene overexpression (*WT1*). It is commonly used as a standardized method in hematology laboratories to obtain a diagnosis and to monitor the kinetics of MRD without inter-laboratory mismatch [18,19]. This technique was shown to be reproducible, accurate and highly sensitive for MRD monitoring, with a significant capacity in predicting prognosis, treatment effectiveness and relapse risk [20] (Figure 2).

In relation to this, the European LeukemiaNet (ELN), a group of 24 international experts in the clinical and translational knowledge in MRD in AML, has established the RT-qPCR approach as “highly sensitive” and as the “gold standard” [21].

ELN indicates that molecular evaluation of *PML-RARA*, *RUNX1/RUNX1T1*, *CBFB-MYH11* and mutated *NPM1* need to be considered as a standard of care for AML patients, and it recommends performing that assessment at diagnosis and every 3 months for 24 months after treatment editing [16]. 

*PML-RARA* is the fusion gene responsible for acute promyelocytic leukemia (APL). Three main alternative transcripts (bcr1, bcr2 and bcr3) may arise according to the location of breakpoints [5]. RT-qPCR has proved to be highly sensitive, readily standardized and to date represent the gold standard analysis for diagnosis and treatment monitoring of APL. MRD follow-up in patients treated with ATRA or ATO is considered important to achieve molecular remission [22]. Furthermore, given the small rates of relapses even in patients with intermediate-risk, the endpoint of treatment (previously associated with PCR negativity) has been substituted with a suggestion to maintain the treatment plan and MRD monitoring in BM until negativity, even if *PML-RARA* levels remain detectable [16]. Monitoring MRD for at least two years after the end of therapy remains mandatory for high-risk APL [16]. The molecular assessment involved the use of a single RT-qPCR protocol, meaning three reactions, one for each *PML-RARA* transcript [18]. This approach proved to be too laborious and costly. Consequently, a 3-plex RT-qPCR assay has been designed for fast molecular diagnosis and MRD monitoring of APL. Composed of multiple primers and probes, it allows seeing simultaneously all three main *PML-RARA* fusion transcripts [23]. 

Core-binding factor acute myeloid leukemia (CBF-AML) is a subtype of AML characterized by *RUNX1/RUNX1T1* translocation or inv(16) and associated with a better prognosis compared to other types of AML [4,5]. RT-qPCR analysis of MRD has proved to be a sensitive and accurate method regarding peripheral blood (PB) levels of *RUNX1/RUNX1T1* after consolidation therapy and during remission and in predicting relapse risk [24]. Jourdan et al. [25] illustrated that 3-log MRD of *RUNX1/RUNX1T1* or inv(16) reduction is useful for discriminating high-risk patients from low-risk patients. However, a recent study investigating the real contribution of measuring transcript kinetics of CBF-AML showed that the majority of relapses were not predicted by molecular monitoring and occurred in a very short period of time, suggesting that MRD monitoring may be poorly informative in the follow-up of CBF-AML patients [26]. Moreover, the permanence of a low amount of positive transcripts in patients with long-term remission without effects on treatment outcome has encouraged the development of more accurate methods, such as ddPCR [23]. 

*NPM1* has an incidence of mutation near to 30–40% of adult AML and is frequently associated with normal karyotype (50–60%). To date, more than 50 different mutations are described [27]. Types A, B and D are the most abundant (approximately 90% of *NPM1* mutated AML patients) [28]. Being one of the most frequent molecular lesions observed in AML, it is an optimal leukemia-specific MRD target, with implications in clinical practice [29]. However, RT-qPCR is limited to patients carrying out types A, B and D since commercial plasmid standards are available for them. 

ELN recommends the monitoring of *NPM1* transcripts in BM and PB. The recurrence of *NPM1* mutation in BM after treatment and the not attainment of a 4-log reduction in PB requires closer monitoring every 4 weeks for at least 3 months [16]. Indeed, even if *NPM1* mutation is classified as a favorable risk, its persistence after the second cycle of chemotherapy was associated with a higher relapse risk and reduction in OS independently of other prognostic factors [30]. Finally, *NPM1* qPCR analysis has proved to be essential as a marker of allogeneic stem cell transplantation in poor responders [31,32]. 

Nevertheless, RT-qPCR has some limitations: (1) more than 60% of AML patients cannot be analyzed by RT-qPCR because they lack common MRD molecular targets; (2) RT-qPCR needs a plasmid standard curve for each molecular target analysis, limiting its usage; (3) identification of the most clinically significant time-points and MRD thresholds; and (4) detection limit in monitoring AML patients with long term remission, where the level of fusion transcript is extremely low. These difficulties can interfere with risk assessment, and they have driven to development of droplet digital PCR (ddPCR), a recently designed technology that can reach high-precision absolute quantification (Figure 2). 

## 3. Digital PCR: Emerging Approach for Diagnosis and Follow-Up in Myeloid Malignancies

Digital polymerase chain reaction (dPCR), or more precisely called droplet digital polymerase chain reaction (ddPCR), is a superior adaptation of real-time quantitative polymerase chain reaction (RT-qPCR). Since its first use in 1999 to detect *ras* mutations in colon cancer patients, it has been used primarily to detect mutations in genes linked to cancer genesis [33]. Although not a new method, dPCR has only recently entered the focus of scientific interest. Its field of application is increasing, not only in basic science but also in clinical diagnosis [34,35,36]. As with the classical PCR method, dPCR uses the same primer sets and fluorescent and enzymatic reagents. The difference is that the sample of interest is divided into thousands of individual PCRs-compartmentalisation, creating an environment with limited dilution. This eliminates the need to create a standard curve and achieves high precision and sensitivity [33]. Then, at the end of the PCR, fluorescence is measured (end-point PCR). Furthermore, Poisson statistics are used for data analysis. Likewise, ddPCR operates on the principle of fractionation of the sample of interest at about 20,000 droplets in an aqueous-oil emulsion, followed by a PCR reaction on each individual droplet [37].

The technical similarities and differences between dPCR and RT-qPCR are well defined, and the most important ones are listed as follows: (i) dPCR consists of thousands of individual PCR reactions divided during analysis while in RT-qPCR there is only one exponential amplification of the nucleic acids; (ii) dPCR measures the absolute number of the molecule of interest, while RT-qPCR determines the relative number using a standard curve; and (iii) dPCR has higher sensitivity and better accuracy compared to RT-qPCR [38,39]. The attractiveness of dPCR also increases with the fact that it does not require the use of standard reference curves. Given the existence of specific genetic aberrations in most hematological neoplasms, dPCR is increasingly widely used to make an accurate diagnosis of hematological diseases and to obtain pathological gene quantification [40,41,42]. 

Nevertheless, the genetic complexity and molecular profile of AML complicates the decision to select a factor for determining MRD, which in turn is necessary for better risk stratification and therapy selection. In addition, the different approaches available for determining MRD have not been the subject of international standardization to date. Thus, monitoring patients with AML and early detection of possible relapse remains a major clinical challenge [10,16]. In this regard, the emerging dPCR approach is increasingly used in the detection of somatic mutations and the determination of MRD among AML patients [43]. The subclonal heterogeneity of AML requires the combined detection of multiple mutations simultaneously for better insight into disease progression and more recently for the implementation of the new target therapeutic agents. 

In general, proving the presence of *NPM1* mutations and the fusion genes (*RUNX1-RUNX1T1*, *CBFB-MYH11* and *PML-RARA*) by using the dPCR technique and then evaluating them after induction and consolidation chemotherapy are suitable approaches for molecular MRD assessment [16]. There are several other somatic mutations that can be considered for MRD monitoring in AML, such as *DNMT3A*, *NRAS*, *ASXL1*, *TET2*, *FLT3-ITD*, *FLT3-TKD*, *IDH1*, *IDH2*, *WT1*, etc. A recent study evaluated the use of a newly developed double drop-off droplet dPCR (DDO-ddPCR) assay for mutation screening and AML disease monitoring using cell-free DNA [43]. In the study, assays were developed to determine the following mutations: *NPM1* (a marker of residual disease) and *IDH2* (therapeutic target for enasidenib). In conclusion, this new dPCR assay offers rapid diagnosis and detection of somatic mutations in AML patients, crucial for a prompt and targeted therapeutic decision, and then facilitates disease monitoring. The cost–benefit compared to other modern diagnostic methods such as NGS is on the dPCR side in terms of price, availability and sensitivity. 

Several other studies have analyzed the *IDH1/IDH2* mutation as an appropriate marker for molecular monitoring in AML using dPCR [44,45,46]. Given the frequent presence of these mutations in the molecular profile of AML as well as the development of new target therapies, *IDH1/IDH2* will be the focus of future scientific research. dPCR can also be used to quantitatively assess the WT1 molecular marker in AML patients with high accuracy [47]. Its importance in disease follow-up has been recognized for years, and the RT-qPCR method has been standardized for its determination [48].

Finally, allogeneic hematopoietic stem cell transplantation (HSCT) as a curative method remains one of the greatest successful hopes over AML. Balsat et al. have shown that the presence of MRD prior to or after HSCT is associated with a negative outcome [32]. Currently, introducing a method into routine clinical practice with the capacity for early prediction of disease relapse is becoming an increasingly desirable goal for therapy. Indeed, eradication of MRD immediately preceding transplant increased the opportunity of long-term survival in AML [49]. dPCR as a method for assessing MRD has proven to be effective and highly trusted for the detection of pre-transplant NPM1 mutation burden and, thus, for the prediction of relapse in AML patients [18,50]. In another study, dPCR proved to be a more effective method of predicting disease relapse than RT-qPCR regarding the time frame [51]. 

The scientific data so far place dPCR at the zenith of interest when it comes to hematological diseases, especially where we need long-term monitoring. Its reliability as a method with high sensitivity and precision, most importantly in low levels of the disease, has been confirmed in numerous studies. The fact that, unlike RT-qPCR, there is no need for a standard curve and reference material makes it attractive, easier to access and fast to use. However, the main disadvantage of dPCR in monitoring AML patients is that it requires a specially designed assay for each specific gene aberration. 

## 4. Next Generation Sequencing (NGS): Unveiling of the Molecular Landscape in Myeloid Neoplasms 

NGS or massively parallel sequencing is a revolutionary method of DNA and RNA sequencing. It is called parallel because it sequences millions of DNA fragments simultaneously. Sequencing may be limited to selected segments of certain genes or to the entire exome [52]. The workflow that NGS runs is as follows: (i) library preparation, (ii) sequencing and (iii) data analysis. In the first stage, the DNA or RNA sample is prepared for sequencing, while fragmenting and adding special adapters to both ends of the fragments. In this manner, the fragments can be amplified. In the next step (ii), the fragments are placed in a flow cell and sequencer. The clusters of DNA or RNA fragments are then amplified in a process called cluster generation, creating millions of copies of single-stranded DNA or RNA. In this very step, chemically modified and fluorescently labeled nucleotides, through the principle of natural complementarity, bind to the DNA template and prevent the next base incorporation. The last step (iii) is data analysis, i.e., the determination of incorporated nucleotides [53].

In the early years of its appearance, NGS platforms were used primarily for cancer research purposes. Recently, they are increasingly emerging as irreplaceable diagnostic tools in clinical settings. So far, there are several commercially available NGS myeloid panels. They target about 30 genes directly or indirectly involved in the pathophysiology of myeloid neoplasms. Depending on their function, these genes may belong to the group of transcription factors, epigenetic modifiers, signal molecules, etc. 

The clinical use of NGS proved to be important in demystifying myeloid neoplasms that lack classical chromosomal or gene aberrations. For example, in Ph-MPNs, in addition to the presence of “driver” mutations, the application of NGS in a clinical setting reveals new mutations that facilitate risk stratification and treatment decision [54]. NGS reveals an entirely new concept of disease understanding, pushing several layers deeper into the genetic profile and directly opening the possibility for new therapeutic approaches. The co-occurrence of newly discovered and “driver” mutations also offers a new concept regarding prognosis [55]. Of particular interest here is the detection of a mutation that will predict early progression (development of) in secondary AML. Thus, the co-existence of *ASXL1*, *SRSF2*, *EZH2*, *IDH1* and *IDH2* in PMF patients is associated with shorter leukemic-free survival and an increased risk of leukemic transformation [54,56]. Similarly, in ET and PV patients, mutations in the *IDH2*, *U2AF1*, *EZH2*, *TP53*, *SH2B3* and *SF3B1* genes are associated with a worse prognosis [57]. NGS enables more detailed detection of each patient’s molecular map and further efficient selection of HSCT candidates. 

The idea of MRD assessment with NGS in AML patients has existed since the very beginning of NGS expansion in clinical settings. Scientific studies have shown that 96% of AML patients have at least one driver mutation, and 86% have at least two [58]. By improving clinical applicability and increasing sensitivity, NGS can be a valid tool for MRD assessment in AML patients, especially among those with rare gene mutations. One study, based on mutation detection in *NPM1* and *FLT3-ITD* genes, showed that NGS had assured MRD assessment and 95% concordance with RT-qPCR for mutated *NPM1* [59]. 

In a study by Morita et al. using targeted sequencing of 295 genes in 131 AML patients, the lower cumulative incidence of relapse (CIR) and better overall survival (OS) were found among patients who had no residual mutations until 30 days after induction therapy [60]. *RUNX1* gene evaluation with NGS is also a possible choice for MRD analysis in AML patients. In one study in this context, mutational burden <3.61% was associated with better event-free survival (EFS) and OS [61]. In a large study of 482 AML patients using a 54-gene NGS panel, samples were sequenced at the time of diagnosis and in the phase of clinical remission after induction chemotherapy. It was found that, in almost 90% of patients, at least one detectable mutation was present at the time of diagnosis. Using the same assay, the same analysis was performed after therapy, and a mutation was observed in 51% of the patients. A conclusion of great importance in this study is the fact that patients who had only *DTA* mutations (*DNMT3A*, *TET2* and *ASXL1*) had a reduced risk of developing relapse, while patients with persistent mutations in other genes had an increased risk of developing relapse [62]. 

The fact that at least one leukemic mutation is present in a large number of AML patients permits us to believe that any of these mutations may be an appropriate marker for MRD, and NGS can provide an effective MRD assessment. Additionally, NGS can detect reciprocal gene rearrangements such as *PML-RARA*, *RUNX1-RUNX1T1* and *CBFB-MYH11*. The NGS method is particularly superior in detecting intra-chromosomal rearrangements compared to FISH, which can detect these changes with great sensitivity only on larger chromosomes [63]. However, even if NGS can be used to detect MRD markers, association with cytogenetic and PCR-based approaches are essential to quantify correctly the presence of target [64]. At this time, detection of novel mutations or gene variants by NGS is not associated with change in treatment plan since their functional consequences are not yet fully understood. Furthermore, NGS is considered useful to define relapse, but it would be necessary to identify clinically the meaning of novel genetic mutation and their impact on disease patterns.

The future advent of genome-wide approaches in clinical practice could allow the identification of additional driver gene mutations and potential MRD markers suitable for prognosis and innovative therapeutic procedures [65].

NGS offers precise gene sequencing, but what is the true impact of the discovered mutations on leukemogenesis? These questions remain unclear to clinicians and scientists; therefore, one of the imperfections of NGS is the inability to determine the impact of a particular mutation. Hence, the challenge of introducing it into routine clinical practice [54]. Furthermore, a distinction must also be made between leukemia-related somatic mutations and clonal hematopoiesis of indeterminate potential (CHIP). CHIP by definition is a process associated with the aging of hematopoietic cells, in which they form clones that have acquired leukemia-related mutations with an allelic frequency of 2% or more. Thus, in AML patients, even in the period of clinical remission, certain mutations of genes such as *TET2*, *ASXL1*, *RUNX1*, *IDH*, *DNMT3A* and others may be present [66]. Along this line, another important aspect to consider when interpreting NGS assays includes germline mutations in certain genes that may be involved in leukemogenesis. The role of these germline mutations is not always clear; thus, their numbers are likely to grow in the future as NGS progresses. For example, one study reported germline *p53* mutations in 6 of 107 AML patients after cancer treatment [67]. Thus, the role of this mutation in leukemogenesis is undoubtedly clear. 

It will be of great importance in the future to create updated and extensive cancer databases that would include all mutations that can initiate the leukemogenic process. Moreover, NGS analysis in AML patients is of particular importance applied to the *IDH1*, *IDH2* or *FLT3-ITD/TKD* genes, as they may represent a hot spot for target therapy. NGS as a newly introduced method will proceed through many more processes of intensive comparative analysis with existing methods of molecular diagnosis before being applied in clinical practice. A multidisciplinary approach must be taken to overcome technical, economic and organizational aspects.

## 5. Systemic Metabolomic Profiling: The New Era of Personalized Medicine

The qualitative and quantitative collection of cell metabolites including carbohydrates, vitamins and lipids featured in a specific condition or in a common metabolic reaction is defined as metabolome. It is a result of biochemical reactions catalyzed by the proteins of the proteome. Therefore, the metabolome can be considered as the interaction of genome and proteome information where microenvironmental factors can play an active role.

The understanding of metabolome functions in the past resulted in strong contributions in biological research. Roughly, from the 1920s to the 1960s, the “golden age of biochemistry” clarifies most metabolic networks involved in organisms and that are responsible for nutrient use and energy production. Thus, the main processes such as glycolysis, tricarboxylic acid (TCA), urea cycles respiration, glycogen catabolism, oxidative phosphorylation and the supremacy of ATP in energy transfer reactions have been characterized.

The latest scientific findings and the development of “omics” technologies open a modern concept of metabolism as a complex system operating in network with other biological systems. Metabolomics is the analytical and informatic technique used to study the metabolome and to define cell type, tissue, organ or organism metabolomic signatures in a specific condition [68]. Metabolomics studies allowed understanding disease mechanisms, identifying new diagnostic markers and figuring out drug effects and the individual variation in drug response (pharmacometabolomics). In addition, the metabolomic approach could be unlocking massive potentials for characterizing disease states in translational medicine [69]. The metabolomic approach has considerably impacted the pathophysiology of common complex diseases such as, e.g., Alzheimer [70], cardiovascular diseases [71], diabetes [71,72], asthma [73] and many cancers [74]. In this latest field, it offers a good method for the assessment of diagnosis, prognostication and disease monitoring [74]. 

In the last ten years, differential metabolomics approaches have been used to untangle AML and have paved the way to new information crucial for diagnosis, prognosis and target identification, although the complexity of the related technologies does make it easy to use in routine clinical practice. 

Indeed, this approach provides several skills that many laboratories do not manage: the equipment is very expensive, the laboratory staff needs optimal knowledge on biochemical pathways and the samples must be prepared with the optimized and reproducible method in order to avoid bias associated to technical procedures (Figure 2).

By the Nuclear Magnetic Resonance (NMR)-based metabolism approach, significant serum metabolomic differences between different risk subgroups of AML were found. These studies enriched data concerning several metabolic pathways such as glycolysis, TCA cycle, proteins and lipoproteins biosynthesis, metabolism of fatty acids and cell membrane components [75]. All these results confirmed that the NMR-based metabolomics method was useful for rapid AML diagnosis and prognosis.

Being an early and noninvasive area of interest in AML, metabolomics can improve the investigation of a new biomarker. Combining gas chromatography coupled with triple quadrupole tandem mass spectrometry and statistical analysis, Musharaf et al. studied differentiated metabolic alterations in the serum of patients with acute lymphoblastic leukemia (ALL), and AML Fatty acid palmitic, stearic and oleic acid metabolism emerged as deregulated in patients with acute leukemia, thus representing an essential metabolic pathway associated with disease progression [76]. 

Metabolomic profiling by ultra-high performance liquid-chromatography mass spectrometry was used to study the metabolome of FLT3-ITD positive AML [77], which until now has been poorly understood. Using ultra-high performance liquid-chromatography-mass spectrometry, they identified a specific metabolomic signature in plasma and in leukemic cells according to FLT3 status, including lysophospholipid metabolism, cysteine/methionine metabolism, tryptophan metabolism, purine metabolism and biosynthesis and carnitine mediated fatty acid oxidation [77,78,79,80].

Recently, a global untargeted metabolomics approach was used to ascertain sorafenib resistance in FLT3/ITD-mutated leukemic cells [81]. Altered glycolytic activity and TCA cycle, increased antioxidant capacity of GSH and reduction in PPP flux rates result as major modifications between sensitive and resistant groups [81]. A combinatorial transcriptomics and metabolomics analysis provided evidence for additional therapeutic targets that are useful for enhancing the efficacy of gilteritinib, another FLT3 inhibitor. Metabolomic alterations are prevalently associated with decreased glutaminolysis and disruption of redox homeostasis, suggesting new therapeutical strategies for a subset of relapsed/refractory AML [82].

Combining different types of “omics” represents a promising future goal in order to offer personalized therapeutic approaches even for AML patients with identifiable and targetable genomic lesions [7,58,83]. Recent genomic-metabolic study on intracellular and biofluid metabolic AML samples allowed identifying a specific NPM1-mutated AML subgroup with high levels of serum choline, trimethylamine-N-oxide and leucine, characterized by common mutations of genes involved in DNA damage response and/or chromatid cohesion (NPM1/cohesion-mut) pathway. These data provided a pattern of the crosstalk between metabolic and genomicspathways in AML. These results, highlighted as an integrated genomic-metabolic study applied in pathology, could pave the way to personalized medicine [84].

## 6. Conclusions

Although there were enhancements in AML diagnosis, classification and remission achievement, more than 50% of patients relapsed due to the persistence of residual resistant clones. Considering this, the new era of the AML study approach passed through a precise and accurate evaluation of residual disease.

The improvement of new techniques such as ddPCR and NGS, attempting MRD detection, completely modified the relevance of this issue; currently, MRD monitoring has become an objective methodology for setting up remission statuses. In this context, the “minimal” residual disease (MRD) term has been substituted with “measurable” residual disease to emphasize the crucial role of modern techniques in determining the threshold of remission and in activating preventive treatment of molecular relapse. In addition to these technologies, metabolome analysis of AML serum highlighted the possibility to identify significant metabolic signatures suitable for the detection of relapsed patients and of highly responding patients with risk stratification. For this issue, standardization of sample selection and processing will be necessary to improve the use of metabolomics in AML stratification. In the near hematological future, characterizing novel drugs for metabolomics targeting and improving personalized treatment approaches will be challenging.

## Data Availability

Not applicable.
